# Editorial: New advancement in network and path-analysis approaches for the study of disorders within the impulse-compulsive spectrum disorders

**DOI:** 10.3389/fpsyt.2022.1029122

**Published:** 2022-09-26

**Authors:** Roser Granero, Isabel Krug, Susana Jiménez-Murcia

**Affiliations:** ^1^Department of Psychobiology and Methodology, Universitat Autònoma de Barcelona - UAB, Barcelona, Spain; ^2^Ciber Fisiopatología Obesidad y Nutrición (CIBERobn), Instituto Salud Carlos III, Madrid, Spain; ^3^Psychoneurobiology of Eating and Addictive Behaviors Group, Neuroscience Program, Institut d'Investigació Biomèdica de Bellvitge - IDIBELL, l'Hospitalet de Llobregat, Spain; ^4^Melbourne School of Psychological Sciences, The University of Melbourne, Melbourne, VIC, Australia; ^5^Department of Psychiatry, Hospital Universitari de Bellvitge, L'Hospitalet de Llobregat, Spain; ^6^Department of Clinical Sciences, School of Medicine, Universitat de Barcelona - UB, L'Hospitalet de Llobregat, Spain

**Keywords:** impulsive-compulsive disorders spectrum, psychiatric disorders, network analysis, path-analysis, impulsivity

## Conceptualization of the impulsive-compulsive disorders spectrum

The reference taxonomies in the clinical and research settings [the *Diagnostic and Statistical Manual of Mental Disorders* DSM-5 (1) and the *International Classification of Diseases* ICD-11 (2)], define the impulsive-compulsive disorder spectrum (ICDS) as a range of diverse separated diagnostic categories. These categorical classifications are essentially based on the classical biological model, consider each disorder within the ICDS as a different nosological unit, and assume the existence of a latent underlying process as the cause of the pattern of symptoms (described in a criteria list). The rationale for defining the ICDS as a group of distinct symptom structures and clinical endo-phenotypes is the presence of repetitive behaviors (in some cases ritualistic actions) with reduced control to inhibit actions, even in the presence of severe negative consequences and harms (3, 4). Patients within the ICDS perceive their problematic behaviors as urgent and irresistible, and experience pleasurable feelings while carrying out these actions (5). Other common characteristics are age of onset variation between childhood to young adulthood, fluctuating lifelong course, extensive impact on the quality of life, and comorbid associations with diverse psychiatric conditions. Common neurobiological processes have also been associated to the onset and the progression of the diverse disorders within the ICDS (6), such as changes in the dopaminergic system of a mesocorticolimbic circuit, the so-called reward system (which involves different areas in the midbrain).

Epidemiological studies warn that clinical conditions under the ICDS may be underdiagnosed within clinical and population-based samples. The complex health states and the multiple inter-related symptoms could not be spontaneously reported by patients (because they perceive shame or fear due to the social stigma, or even because they do not know that the symptoms can be effectively treated) (7–9). On other occasions the symptoms identified are attributed to the presence of a concrete disorder, and likely comorbid conditions are not assessed (and therefore, neglected).

## New methodological research approaches for the study of ICDS

The empirical evidence sustaining separate categorical taxonomies grouped within the ICDS is largely based on statistical procedures such as general/generalized linear models and classification procedures (cluster analysis and latent class analysis). But arguments against classical categorical taxonomies exist, and alternative problem-oriented systems support that the diverse endo-phenotypes grouped into wide ranges such as the ICDS are better embedded through myriads of multivariable inter-connected systems. These alternative models contemplate mutually interacting-dynamic biological, psychological, inter-personal and contextual impacts on the individuals' health. In addition, these alternative systems suppose that boundaries between diagnostic entities may be not clearly defined, and that these fuzzy limits are better explained through the presence of potential transdiagnostic characteristics and/or patterns of symptoms (10). Path-analysis and network analysis are statistical approaches that can contribute to the development of these new diagnostic formulations, through powerful tools for examining and visualizing complex structures in a more realistic way than classical procedures (11).

Path-analysis is aimed to examine a set of simultaneous relationships between variables and estimating the magnitude and significance of direct and indirect effects (meditational links). This approach has been historically used to test a theoretical model with *causal* relationships between variables (12). But current studies use path-analysis for both exploratory and confirmatory modeling, and therefore it is now employed to theory testing and theory development (13). Path-analysis is usually implemented as a case of structural equation modeling (14), and it is visualized through path-diagrams. The model specification (rationale for the underlying paths) is based on the cumulate empirical evidence.

Network analyses employ tools from Graph Theory aimed to characterize spatial/functional structures between the nodes (15–17), which represent the patterns of relationships among symptoms (18). In medical clinical research, network structures are visualized through diagrams based on nodes (plotted as circles which are the representation of the symptoms and/or other features of the endo-phenotypes) and edges (plotted as connector-lines which characterize the associations between the nodes) (19–23). Concepts related to the network analysis are centrality (identification of the key symptoms associated to the onset, the severity and the progression of the clinical profiles) and closeness (identification of the linkage or transition capacity of the nodes, particularly useful for the study of the pathways to comorbid conditions) (22, 24–27).

## Motivation for this Research Topic

The manuscripts included in this Research Topic provide new empirical evidence (obtained through diverse methodological approaches (including path-analysis and network analysis), that contribute to a better understanding of the onset, the progression, and the structure of the diverse endo-phenotypes within the ICDS.

One study of this Research Topic is aimed to test the validity of the neuroscientific theory on the dominance of compulsivity over impulsivity in severe cases of behavioral addictions, including gambling disorder, internet gaming, exercise dependence, compulsive buying, and hair-pulling (Demetrovics et al.). The data collected from a large population-based sample (*n* = 2,710) show that compulsivity dominates over impulsivity in the more severe symptomatic states of the behavioral addictions, which could be interpreted as a shift from reward-driven to relief-driven and habitual behavior in the disease course. This process seems congruent with findings in animal models of addiction based on neuroscientific evidence.

Other work of this topic focused on the incentive sensitization theory is aimed to test the dissociation between “wanting” and “liking” across different substance related disorders (including alcohol, nicotine, cannabis, and other drugs) and addictive behaviors (gambling disorder, overeating, gaming, pornography use, sex, social media use, Internet use, TV-series watching, shopping, and work) (File et al.). The results of a path-analysis provide support about the role of incentive sensitization in both potentially problematic substance use and behavioral addictions. Interesting, the structural equation model also suggests that impulsivity might not be directly associated with problematic engagement in substance use and problematic addictive behaviors, but indirect impacts emerge through the meditational role of “wanting.”

A third work in this Research Topic uses path-analysis for assessing the associations between diverse impulsivity measures (including self-reported, behavioral data and the experiential discounting task) with compulsivity measures (registered through neuropsychological tools) (Mestre-Bach et al.). The results obtained among a clinical sample of gambling disorder patients (*n* = 132) show that the gambling severity levels are differently explained by the diverse aspects of impulsivity and compulsivity. And this specific outcome reinforces the idea that impulsivity and compulsivity constitute two multifactorial constructs with distinct impacts on the gambling profiles.

Other three studies included in this Research Topic are aimed to obtain evidence regarding the underlying relationships among a set of symptoms covering different constructs related to the ICDS. The manuscript of González-Bueso et al. obtains differences in the sexual behaviors and other sociodemographic and clinical variables between patients with online-compulsive sexual behavior (OCSB, *n* = 36) vs. non-online compulsive sexual behavior (non-OCSB, *n* = 44). Compared with a healthy control group, both clinical sub-samples show worse psychopathology state, higher harm avoidance and self-transcendence levels, and lower self-directness and cooperativeness. Compared to OCSB, non-OCSB patients also exhibit higher likelihood of sexually transmitted diseases, higher odds of homosexual and bisexual orientation, higher anxiety levels and more difficulties in sexual impulse control.

The study of Reivan Ortiz et al. uses path-analysis for testing a meditational model explaining the perfectionism level among a clinical sample of patients who met diagnostic criteria for anorexia nervosa, bulimia nervosa and obsessive-compulsive disorder (*n* = 187). The results of this work show a direct effect of the preoccupation with errors and goals manage on the perfectionism score, as well as an indirect effect mediated by the emotion (dys)regulation severity. In addition, invariance by the diagnostic subtype is achieved, and this concrete outcome is interpreted as evidence about the role of the perfectionism construct as a non-adaptive criterion that exacerbates the symptom level related with eating disorders and obsessive-compulsive disorders.

The last manuscript of this Research Topic, the study of Cascino et al. investigates the association of the eating disorders key symptoms and other psychological measures (including aggressiveness) through network analysis, among a clinical sample of women with bulimia nervosa (*n* = 69). In this work, interceptive awareness and ineffectiveness are the nodes with the highest centrality in the network composed by the eating disorder symptoms, while guilt is the only negative emotion with bi-directionally associations within the eating problems. Likewise, verbal hostility and resentment are the highly central nodes in the network profile defined by the hostility measures. And the variables with the higher bridge strength centrality in a network defined with the complete phenotype were impulsivity, drive for thinness, ascetism, guilt and hostility.

## Conclusion

The results obtained in the studies collected in this Research Topic are consistent with previous research, which suggest the relevant role of impulsivity within ICDS. Impulsivity constitutes a complex multifaceted psychological construct, strongly related with the difficulties in the control of thoughts and behaviors: individuals with high impulsivity levels show speedy responses with low reflection, despite possible negative consequences and harms (28). Impulsivity has also been conceptualized as an endophenotype of vulnerability and a transdiagnostic feature, characterized by patterns of risky health-related decision-making, that can be displayed as a temporary state and/or a generalized trait over time and across contexts that may contribute to multiple psychiatric disorders (29, 30). For example, a current systematic review and voxel-based meta-analysis focused on the relationships between brain morphology and trait impulsivity, has identified impulsivity-related volumetric gray matter alterations in prefrontal, temporal, and parietal cortices, as well as interaction effects of age and gender (31). Within the ICDS, neurobiological basis of impairment in some executive functions have been related to behavioral inhibition and impulsivity (32). Adequate executive functioning is a requisite to achieve an adequate adaptation to the environment and its demands, and includes diverse multiple neurological processes located in the prefrontal lobes and subcortical connections: reasoning, planning, decision-making, organization, flexibility, inhibition, working memory, and monitoring. Compared to healthy controls, ICDS patients have been found to exhibit deficits on neurocognitive domains involving attention, verbal and visual memory, visual-spatial abilities, shifting capacity, top-down control, and flexible responding (33, 34). Moreover, neuroimaging studies have observed changes in the activity in the orbitofrontal cortex and ventral striatum in discounting tasks (a measure of the impulsivity related to disinhibition and impaired reward sensitivity). Compared to healthy-control samples, patients within ICDS exhibit increased risk taking/probability discounting (e.g., choosing larger but less probable rewards instead of smaller but more probable ones), and altered delay discounting (e.g., choosing immediate smaller rewards over delayed larger ones) (35, 36).

Studies collected in this Research Topic also provide evidence regarding the different role of the compulsive vs. the impulsive processes in the disorders within the ICDS, and this precise difference is reflected in the form of a compulsive-impulsive continuum (37). One side of the continuum includes disorders with a compulsive nature (i.e., obsessive-compulsive disorder, anorexia nervosa, body dysmorphic disorder and hypochondria), while the other side of the continuum comprises disorders with an impulsive component i.e., behavioral addictions (i.e., gambling disorder, gaming addiction, compulsive buying-shopping, and sex addiction) and eating disorders with binge-purging symptomatology (i.e., bulimia nervosa and binge eating disorder). Individuals with the compulsive types of disorders normally avoid potential risk and seek security, mainly because they perceive high harm avoidance and are unable to resist the need for decreasing anxiety and distress levels (38). Conversely, the impulsive type of mental illnesses are characterized by the incapacity of patients for adequately estimating the probability and the severity of risky actions, as well as the potential harms and negative consequences of these actions (39).

In relation to the above, deficits in the impulse control processes are characteristics in all the conditions within the ICDS (40, 41), but drive for impulsivity differs amongst the two continuums: while impulsive-repetitive behaviors are the result of risk taking (seeking pleasure) during the initial stages for patients with impulsive related disorders, risk avoidance (decreasing negative mood states, anxiety and distress) is the basis of uncontrolled behaviors among patients with compulsive related disorders (37, 42). The conceptualization of a common spectrum also assumes that the different/opposite systems sustaining compulsive and impulsive behaviors can co-exist in the same patients throughout the progression of the diseases (longitudinal comorbidity) (43). In fact, although impulse related disorders are characterized by the pursuit of gratification or reward, as has been mentioned, in many cases it occurs only in the short term. Moreover, this immediate gratification disappears as the condition progresses (44). Studies also suggest that impulsivity and compulsivity may be considered as a phenomenological and comorbid overlap, in the sense that they both imply core problems with top-down inhibitory control (33, 40).

### Implications

The new evidence-based knowledge revealed by the studies grouped in this Research Topic can contribute to adapt and develop new assessment tools, with high sensitivity to identify the presence of the diverse symptoms and signs connected to the ICDS. Results can also contribute to improve and create precise treatment tools, for a careful monitoring of the multiple behaviors-domains of patients within the ICDS (the interventions should be adapted to the specific needs of each patient).

Other research fields can also contribute to develop new non-invasive quantitative procedures to identify networks of structural and functional brain changes in patient within the ICDS. For example, into the psychoradiology area, an emerging field that applies radiological imaging techniques to the study of mental illness (45, 46), with the aim to provide a new basis for transform the diagnostic task from a set of separate symptom-based syndromes to an imaging-based taxonomy (47). Ultimately, efficient prevention and intervention of ICDS rely in a good understanding of how the diverse pathological processes develop.

## Author contributions

All authors listed have made a substantial, direct, and intellectual contribution to the work and approved it for publication.

## Funding

This editorial was funded from Ministerio de Ciencia, Innovación y Universidades (grant RTI2018-101837-B-100). FIS PI20/132, FIS PI17/01167 received aid from the Instituto Salud Carlos III (Ministerio de Sanidad, Servicios Sociales e Igualdad). The research was also funded by the Delegación del Gobierno para el Plan Nacional sobre Drogas (2019I47 and 2021I031), CIBER Fisiología Obesidad y Nutrición (CIBERobn) is an initiative of ISCIII. We thank CERCA Programme/Generalitat de Catalunya for institutional support. Fondo Europeo de Desarrollo Regional (FEDER) Una manera de hacer Europa/A way to build Europe. RG was supported by the Catalan Institution for Research and Advanced Studies (ICREA-Academia, 2021-Programme). The funders had no role in the design of the study; in the collection, analyses, or interpretation of data; in the writing of the manuscript; or in the decision to publish the results.

## Conflict of interest

The authors declare that the research was conducted in the absence of any commercial or financial relationships that could be construed as a potential conflict of interest.

## Publisher's note

All claims expressed in this article are solely those of the authors and do not necessarily represent those of their affiliated organizations, or those of the publisher, the editors and the reviewers. Any product that may be evaluated in this article, or claim that may be made by its manufacturer, is not guaranteed or endorsed by the publisher.
